# Escherichia coli NGF-1, a Genetically Tractable, Efficiently Colonizing Murine Gut Isolate

**DOI:** 10.1128/MRA.01416-18

**Published:** 2018-12-06

**Authors:** Marika Ziesack, Michiel A. P. Karrenbelt, Johannes Bues, Elena Schaefer, Pamela Silver, Jeffrey Way

**Affiliations:** a Wyss Institute for Biologically Inspired Engineering, Harvard Medical School, Boston, Massachusetts, USA; b Department of Systems Biology, Harvard Medical School, Boston, Massachusetts, USA; Georgia Institute of Technology

## Abstract

The genome of the murine commensal strain Escherichia coli NGF-1 contains a 5.03-Mbp chromosome and plasmids of 40.2 kbp and 8.56 kbp. NGF-1 efficiently colonizes the mouse gut and is genetically tractable. The genome sequence reported here facilitates genetic engineering and research in mouse models of healthy and diseased intestine.

## Abstract (precis)

The genome of the murine commensal strain Escherichia coli NGF-1 contains a 5.03-Mbp chromosome and plasmids of 40.2 kbp and 8.56 kbp. NGF-1 efficiently colonizes the mouse gut and is genetically tractable.

## ANNOUNCEMENT

The gut microbiome plays a key role in health and disease (1). Escherichia coli NGF-1 is of particular interest because it was isolated from a healthy BALB/c mouse from Charles River Labs, colonizes mice efficiently, and can be engineered with complex genetic circuits (2–6) (Table 1). Kotula and colleagues placed a tetracycline-inducible trigger element and a memory element in E. coli K-12 and E. coli NGF-1 and found essentially identical responses to tetracycline treatment *in vitro* and in the mouse gut (2). Riglar et al. showed that engineered NGF-1 was stable in C57BL/6 mice for over 6 months (3). In the work of Certain at al., E. coli NGF-1 survived near a surgical implant, allowing study of persistent infection (4). Kim et al. constructed a communication system which could be observed in the mouse gut, and Ziesack et al. introduced NGF-1 as a member of an engineered consortium into gnotobiotic mice (5, 6).

**TABLE 1:** Applications of NGF-1 using artificial genetic circuits

Application	Engineering^*a*^	Reference
Gut sensor (molecule)	ATC-inducible trigger element; memory element	2
Gut sensor (pathogen)	Tetrathionate-inducible trigger element; memory element	3
Chronic infection sensor	ATC-inducible trigger element; memory element	4
Interspecies quorum sensing	ATC-inducible signaling element; Lux-triggered memory element	5
Metabolite cross-feeding consortium member in the gut	Triple KO of amino acid biosynthetic pathways; methionine overproduction through antimetabolite selection	6

a ATC, anhydrotetracycline; KO, knockout; Lux, luciferase.

To obtain the NGF-1 sequence, a glycerol stock was used to inoculate an LB agar plate for the isolation of single colonies. A single colony was then used to inoculate an overnight culture in LB broth (37°C, with shaking at 220 rpm). Genomic DNA was extracted using the Qiagen DNeasy blood and tissue kit and quantified using a Life Technologies Quant-iT PicoGreen double-stranded DNA (dsDNA) assay kit. The DNA was sheared on a Covaris S2000 machine, and a library was prepared using an Illumina TruSeq kit. Sequencing was done using the Illumina MiSeq reagent kit v2 (2 × 250 bp), and quality filtering, trimming, and filtering of adapter sequences were performed using FastQC with standard settings (7). The resulting 6.6 million paired-end reads with an average length of 207 bp were assembled *de novo* with SPAdes version 3.7.1 (8), using the “careful” option to minimize mismatches in the final contigs. The 64 resulting contigs were filtered for contaminants via a BLAST search against several E. coli genomes using Projector2 (9) and Ragout (10) with standard parameters, resulting in 51 contigs at an average read coverage of 22×. Contig ends were joined by two methods. First, some contig ends were identified that had ends with identical segments that fell below the alignment threshold of the joining software. These joinings were validated by alignment of the joined sequences with sequences of other E. coli strains. Second, in cases such as those where identical rRNA genes prevented inference of continuity between sequences on either side of the repeated element, we hypothesized associations based on other E. coli sequences and then confirmed the association by PCR using unique sequence flanking primers and observation of a DNA fragment of predicted size. One case of sequence ambiguity was attributed to an inverting-phase variation-type element.

The genome was annotated with the Rapid Annotation using Subsystem Technology (RAST) server (11) and the Pan-Genomes Analysis Pipeline (PGAP) (12), followed by manual curation. NGF-1 contains a 5,026,105-bp chromosome and two plasmids, pNGF1-CROD2 (40,158 bp) and pNGF-colY (8,556 bp), encoding 5,218, 57, and 10 genes, respectively. The colicin-producing plasmid may help explain the efficient colonizing ability of this strain.

NGF-1 is similar to E. coli K-12 and murine E. coli strains. Specifically, NGF-1 has 98% nucleotide sequence identity with K-12 and >99% with mouse-derived strains, such as MP1, ATCC 25922, and LF82. NGF-1 is distinct from all known E. coli strains, but its genome is a mosaic of genes known from other strains, plus prophage genes. NGF is a niacinamide auxotroph, likely caused by a sense mutation in the *nadC* gene (13).

In sum, E. coli NGF-1 is both engineerable and able to colonize the mouse gut and other experimentally relevant environments. Knowledge of its genome sequence should facilitate further studies of gut colonization and may facilitate development of living therapeutics and diagnostics.

### Data availability.

This whole-genome sequencing project has been deposited in GenBank under the accession number CP016007. Raw reads are available under the BioProject accession number PRJNA380756.
